# Three-Level Mixed-Effects Logistic Regression Analysis Reveals Complex Epidemiology of Swine Rotaviruses in Diagnostic Samples from North America

**DOI:** 10.1371/journal.pone.0154734

**Published:** 2016-05-04

**Authors:** Nitipong Homwong, Andres Diaz, Stephanie Rossow, Max Ciarlet, Douglas Marthaler

**Affiliations:** 1 Department of Veterinary Population Medicine, College of Veterinary Medicine, University of Minnesota, Saint Paul, Minnesota, United States of America; 2 Department of Animal Science, Kasetsart University, Kamphaeng Saen Campus, Kamphaeng Saen, Nakhon Pathom, Thailand; 3 Vaccines Clinical Research and Development, GlaxoSmithKline Vaccines, Cambridge, Massachusetts, United States of America; University of Parma, ITALY

## Abstract

Rotaviruses (RV) are important causes of diarrhea in animals, especially in domestic animals. Of the 9 RV species, rotavirus A, B, and C (RVA, RVB, and RVC, respectively) had been established as important causes of diarrhea in pigs. The Minnesota Veterinary Diagnostic Laboratory receives swine stool samples from North America to determine the etiologic agents of disease. Between November 2009 and October 2011, 7,508 samples from pigs with diarrhea were submitted to determine if enteric pathogens, including RV, were present in the samples. All samples were tested for RVA, RVB, and RVC by real time RT-PCR. The majority of the samples (82%) were positive for RVA, RVB, and/or RVC. To better understand the risk factors associated with RV infections in swine diagnostic samples, three-level mixed-effects logistic regression models (3L-MLMs) were used to estimate associations among RV species, age, and geographical variability within the major swine production regions in North America. The conditional odds ratios (cORs) for RVA and RVB detection were lower for 1–3 day old pigs when compared to any other age group. However, the cOR of RVC detection in 1–3 day old pigs was significantly higher (p < 0.001) than pigs in the 4–20 days old and >55 day old age groups. Furthermore, pigs in the 21–55 day old age group had statistically higher cORs of RV co-detection compared to 1–3 day old pigs (p < 0.001). The 3L-MLMs indicated that RV status was more similar within states than among states or within each region. Our results indicated that 3L-MLMs are a powerful and adaptable tool to handle and analyze large-hierarchical datasets. In addition, our results indicated that, overall, swine RV epidemiology is complex, and RV species are associated with different age groups and vary by regions in North America.

## Introduction

Rotaviruses (RVs) belong to the family *Reoviridae* and contain 11 segments of double stranded RNA (dsRNA) [1, 2]. RVs are classified into nine species A-I (RVA-RVI) based on sequencing of the viral protein 6 (VP6) [1, 3, 4]. RVs are a major cause of diarrhea in pigs, and five (RVA-RVC, RVE, and RVH) out of the nine species have been found in swine [5].

RVA is considered the most prevalent, pathogenic, and the major cause of diarrhea in pigs [6]. Early studies indicated that 53% of suckling piglets and 44% of weaned pigs were infected with RVA without evidence of any viral shedding after 2 months of age. In addition, sows infected with RVA were able to shed many different viral strains [7–10]. While the pathogenesis of RVB was established in the 1980s, the revelation of RVB as an important enteric pathogen in pigs was only recently discovered in the United States of America (USA) [11, 12]. RVC were first identified in swine and is an important cause of diarrhea in piglets in the USA [5, 13, 14]. The pathogenesis of swine RVE was established in gnotobiotic pigs although its complete characterization as a RV species is unknown [13]. While the pathogenesis associated with swine RVH is undefined, swine RVH was first identified in Japan and has been recently found circulating in USA and Brazil [15–17]. Co-infections of RVA, RVB, and RVC are common in nursery piglets from the USA while a limited number of co-infections for RVA and RVC have been investigated in other countries [6, 18, 19]. In addition, multiple RV infections can occur within a single swine herd [20], and clinical signs may vary between herds due to strain diversity and/or virulence [21].

Multilevel modeling has been widely used for statistical analysis for more than 50 years [22]. Multilevel modeling incorporates hierarchically demographic information (level) into a single analysis and provides more accurate estimates of effects than conventional fixed-effects modeling. In addition, multilevel modeling allows for multiple comparisons within each level by accounting for the variability within each level [23]. In veterinary epidemiology, multilevel modeling has been used in numerous research investigations involving studies of risk factors for, diarrhea in lambs [24], pre-weaning mortality in goats [25], gastrointestinal diseases in mink [26], Salmonellosis in poultry [27], effects of ketosis on milk production and reproductive problems in dairy cows [28, 29], mortality in sows [30], weaned-to-service interval related to seasonal changes in female pigs [31], and deaths related to seasonal changes in peripartum pigs [32].

The Minnesota Veterinary Diagnostic Laboratory (MNVDL) at the University of Minnesota College of Veterinary Medicine is a large-scale diagnostic laboratory and receives swine samples from North America to identify RV infections. These samples include hierarchical data, which allows for multilevel modeling to estimate the association between RV detection and demographic traits (age, state, region, and country). Currently, three major swine production regions in the USA: Midwest, Southeast, and South-central [33, 34]. Historically, most swine production systems in the USA were located in the Midwest. After the 1980s, swine populations increased in the Southeast (North Carolina and South Carolina) and the South-central (Oklahoma and Texas) regions, and weaned pigs (21 days of age) are transported to the Midwest and raised until their ready for harvest (5–6 months) since the Midwest is the major producer of the feed supply, corn [34].

In the USA, the associations (odd ratios) for swine RVA, RVB, RVC infections are lacking for different pig age groups as well as the relationship of these infections among the different production regions. Therefore, the objective of this study was to investigate the associations among age, RV detection, and regions within the US swine production in samples submitted for diagnosis to the MNVDL.

## Materials and Methods

### Ethic statement

The MNVDL receives animal samples voluntarily submitted by veterinarians or producers in order to determine the causative agent of disease. The MNVDL was not involved in the collection or sampling of the pigs in this study. The MNVDL retains ownership of the samples upon arrival and maintains client(s) confidentiality in public communications by removing any signifiers that would identify the client(s). Client consent is not required if the aforementioned conditions are met.

### Samples and RV detection

The MNVDL received 7,508 swine samples between November 2009 and October 2011 to determine the etiological agent of disease from North America continent. Samples were tested by real time reverse transcriptase polymerase chain reaction (RRT-PCR) for swine RVA, RVB, and RVC using methods described elsewhere [5, 6]. In addition, samples were categorized into five age groups (1–3 days; 4–21 days; 22–55 days: > 55 days; and unknown age) and five geographical regions (Midwest: Illinois, Indiana, Iowa, Kansas, Michigan, Minnesota, Missouri, Nebraska, Ohio, South Dakota, Wisconsin; South-central: Oklahoma, Texas; Southeast: North Carolina, South Carolina; other US states: North Dakota, Pennsylvania, Colorado, Arizona, Alabama, Arkansas, Florida, Kentucky, Tennessee, Utah, Virginia, Vermont, Wyoming, depending on swine production density; and non-USA regions (Mexico and Canada) [35]. The Midwest, South-central, and Southeast represent the densities regions of swine production.

### Statistical analysis and model selection

The RV infectious statuses: RVA, RVB, RVC, RVAB (A+B), RVAC (A+C), RVBC (B+C), and RVABC (A+B+C) were defined for each sample as binary outcome (positive or negative). Tabular methods were performed to calculate and map the frequency distribution of RV status by age group and region with the R packages maps [36], maptools [37], RColorBrewer [38] and classInt [39]. In addition, percentages of RVA, RVB, and RVC were calculated to investigate seasonality of RV infections using a Locally Weighted Regression model [40]. Graphics were produced with the R package ggplot2[41]. Statistical analyses were performed the R version 3.2.2 with different packages as aforementioned [42].

Age, as a continuous independent (predictor) variable, was checked for Normality using Kolmogorov-Smirnov test. To avoid linearity assumption of the independent variable, age was categorized into four main groups; (Age1: 1–3 days, Age2: 4–20 days, Age3: 21–55 days, and Age4: >55 days) (S1 Table). If age was missing with the sample, it was categorized as an Age9: “unknown age group”. The crude associations between RV status and age groups (Model 1) or regions (Model 2) were measured using univariate logistic regression models, and the 1–3 day age group and non-USA region were used as reference group for each model, respectively. Each logistic regression model assumed that each observation (Y_1_,…,Y_7508_; Y_j_) was independent of positive or negative result. Model 1 assumes Y_i_~ Bernoulli (π1); Logit (π1| Age1_i_, Age2_i_, Age3_i_, Age4_i_, Age-unknown _i_) = β_1_ +β_2_Age2_i_+β_3_Age3_i_+β_4_Age4_i_+β_5_Age-unknown_i_. Model 2 assumes Y_i_~ Bernoulli (π2); Logit (π2|Midwest_i_, South-central_i_, Southeast_i_, Other USA-states_i_, non-USA_i_,) = β_1_ +β_2_Midwest_i_+β_3_South-central_i_+β_4_Southeast_i_+β_5_non-USA_i_. The β_1_ is intercept term while β_2_, β_3_, β_4_ and β_5_ are logistic regression coefficients, and π1 and π2 are the probability of being RV positive in the Models 1 and 2, respectively. Because the overall p-value was <0.05 in the univariate logistic regression models, all the age and region covariates were included in the mixed-effects logistic regression models.

Due to the hierarchical structure of the data, three-levels mixed-effects logistic regression models (3L-MLMs, Models 3–5) were performed to investigate the association of age groups on RV detection, and the unknown-age group was excluded from the 3L-MLMs analyses. Because the objective of our study was to investigate associations of age, age was a fixed effect while individual samples (*i*), states (*j*), and region (*k*) were random effects [43–45]. The 3L-MLMs assumed that observations (Y_111_,…,Y_ijk_) are independent of positive or negative result, and Y_ijk_~ Bernoulli (π_ijk_), where π_ijk_ are probabilities of positive results for individual samples (*i*), states (*j*), and region (*k*); hence; Logit (π| Age1_i_, Age2_i_, Age3_i_, Age4_i_, Region_k_, U_k_, W_jk_) = β_1_ +β_2_Age2_ijk_+ β_3_Age3_ijk_ +β_4_Age4_ijk_+ β_5k_Region_k_+ β_2k_Age2_ijk_:Region_k_ + β_3k_Age3_ijk_:Region_k_ + β_4k_Age4_ijk_:Region_k_ +U_k_+W_jk_. The grand mean (β_1_) is the intercept term. The β_2_, β_3_, and β_4_ are the fixed-effects logistic regression coefficients corresponding to the three age groups while β_5k_ are the fixed-effect coefficients at the regional levels (region was assigned to both fixed and random effects in the 3L-MLMs). The β_2k_ β_3k_, and β_4k_ are the fixed-effect coefficients for interactions between the three age groups for the regional levels. The random intercepts U_k_ and W_jk_ were assumed independent across regions (*k*) and across states (*j*) within-the same region *(k)*. The i = 1,…I_j_ are the level 1 indicator for the individual samples (*i)*, j = 1,…J_k_ are the level 2 indicator for the states *(j)*, and k = 1…, K are the level 3 indicator for the region (*k)* (K = 5, J_1_ = 12, J_2_ = 2, J_3_ = 2, J_4_ = 13, J_5_ = 5). Model 3 (the full model) include interaction term between age and region while Model 4 exclude the interaction term. Finally, Model 5 excluded region as a fixed component. The model with the lowest pseudo-Akaike Information Criterion (pseudo-AIC) was preferred as the final model (Model 5). The random effects for regions and states were tested using Likelihood Ratio χ^2^ test (LR χ^2^), which were obtained from residual log-Pseudo-Likelihood using the COVTEST function, and the conditional odds (cOR) of RV detection by age groups were compared to the predefined baseline group (1–3 days old).

For the final model, the variance components were considered random effects and partitioned into three sources level (L1-L3). The L1 variance equals π^2^/3 on the logit scale, the error variance of the binary models [45–47]. The L2 variance equals W_jk_~ *N*(0,τ^2^), the random intercept varying over the effect of states (*j)* (USA or non-USA variance) with zero means and variance τ^2^. The L3 variance equals U_k_~*N*(0,γ^2^), and the random intercept varying over the effect of the region (*k)*, with zero means and variance (γ^2^). The residual intra-class correlation coefficients were estimated to measure dependence and heterogeneity (variation explained by regions) in the three-levels random intercepts (L1-L3). Consequently, the first residual intra-class correlation is defined as *ρ*(region) = γ^2^ / (γ^2^+τ^2^+π^2^/3) with the same region (*k)* but different states (*j)* while the second the residual intra-class correlations is defined as *ρ*(states, region) = (γ^2^ +τ^2^)/(γ^2^+τ^2^+π^2^/3) with the same states (*j)* [43, 45].

Univariate analyses were performed with SAS 9.4, and the 3L-MLMs analyses were performed with PROC GLIMMIX (SAS Institute Inc. Cary, NC). The associations were considered significant when the p-value < 0.05.

## Results

**Fig 1:**
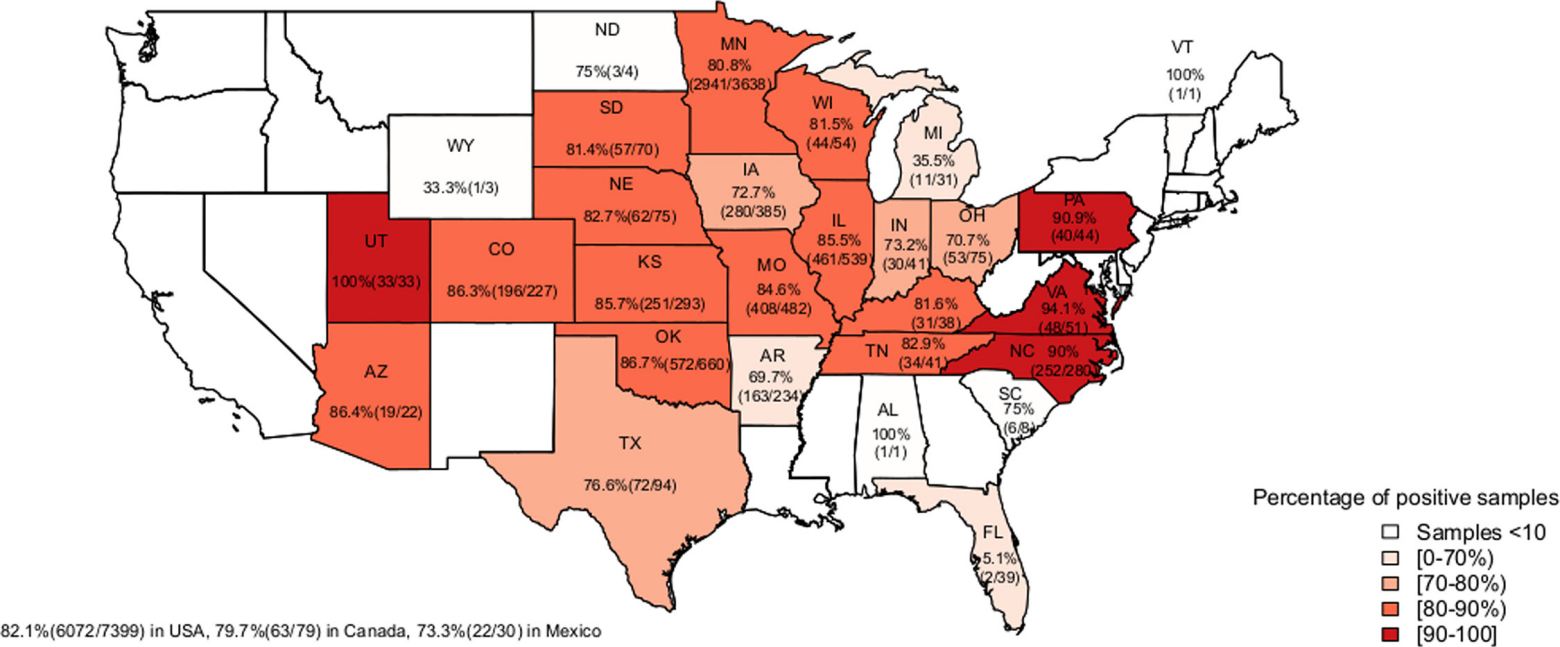
Percentages of any RV positive samples by state. The shading represents variation in percentage of positive samples.

**Fig 2:**
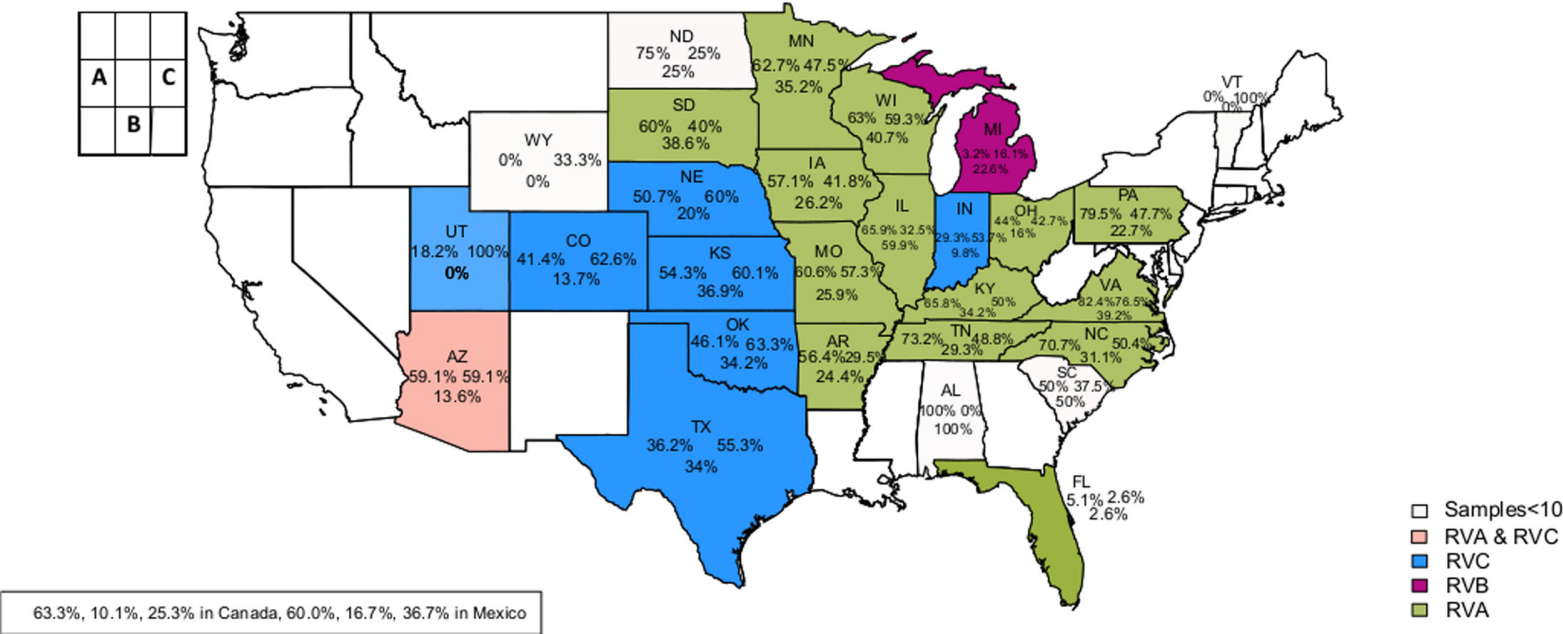
Percentages of RVA, RVB, and RVC samples by state. The color represented highest prevalence of the RV species (green represents RVA, purple represents RVB, blue represents RVC while pink represents equal percentages of RVA and RVC.

**Fig 3:**
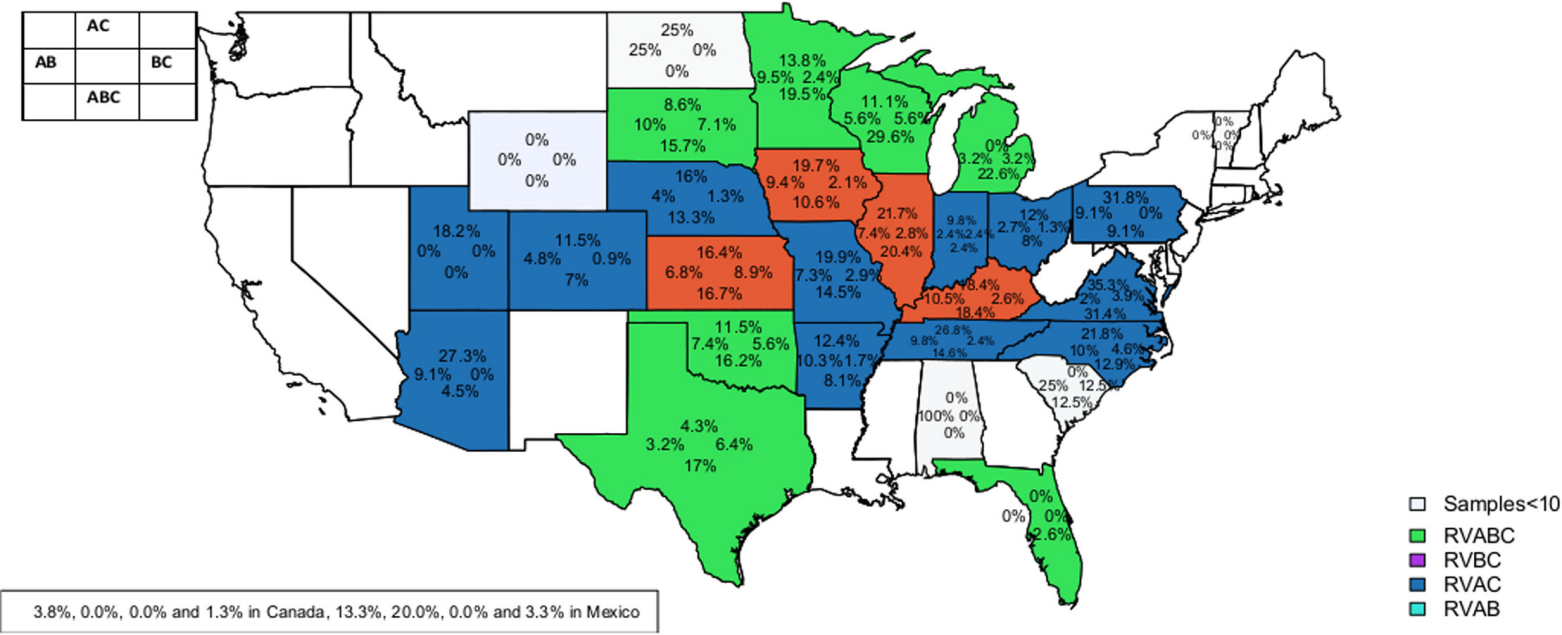
Percentages of positive RVAB, RVAC, RVBC and RVABC sample by state.

In this study, 6158 of the 7508 diarrheic swine stool samples (82.0%) tested positive for RVs. The percentage of positive RV samples from the USA, Canada, and Mexico was 81.1% (6072/7399), 79.9% (63/79), and 73.3% (20/30), respectively. Of the 6072 USA samples, 3638 (59.9%) were from Minnesota, and 2941 (81%) of these samples were positive for RVs (Fig 1). The percentage of RV positive samples by states ranged from 5.1% to 100.0%, with the lowest and highest percentages found in Florida and Utah, respectively. The percentage of RVA positive samples was higher than RVB and RVC in 14 states (Fig 2). Michigan was the only state to have higher percentage of RVB detection while seven states had higher percentage of RVC detection than RVA and RVB. Interestingly, RVA and RVC positive samples occurred in the same percentage (59%) in Arizona. Co-infections of RVAC were detected in the highest percentage in 12 states (Fig 3). Co-infections of RVABC were detected in the highest percentage in seven states while co-infections of RVAB or RVBC were not dominant in our data set.

Samples were categorized into 5 regions; Midwest (n = 5590), South-central (n = 754), Southeast (n = 288), other USA (n = 767), and non-USA region (n = 109) (Table 1). While the highest proportion of RVA positive samples (70.1%) was found in the Southeast region, the highest proportion of RVB and RVC samples were found in the South-central region (34.2% and 62.2%, respectively). Moreover, the highest co-detection of two different RV species was found in the Southeast (21.2%, RVAC). Of the RVABC co-infections, 18.1% of the samples from the Midwest were positive.

**Table 1:** Descriptive statistics of RV positive samples by regions.

Region^2^	Positive for Rotavirus, N (%)								^ ^
	Any RV	A	B	C	AB	AC	BC	ABC	Total^1^
Midwest	4544 (81.3%)	3435 (61.5%)	1851 (33.1%)	2801 (50.1%)	487 (8.7%)	870 (15.6%)	158 (2.8%)	1014 (18.1%)	5590
South-central	644 (85.4%)	338 (44.8%)	258 (34.2%)	469 (62.2%)	52 (6.9%)	80 (10.6%)	43 (5.7%)	123 (16.3%)	754
Southeast	258 (89.6%)	202 (70.1%)	91 (31.6%)	144 (50%)	30 (10.4%)	61 (21.2%)	14 (4.9%)	37 (12.9%)	288
Other-USA	626 (81.6%)	422 (55.0%)	175 (22.8%)	387 (50.5%)	58 (7.6%)	123 (16.0%)	15 (2%)	81 (10.6%)	767
Non-USA	86 (78.9%)	68 (62.4%)	13 (11.9%)	32 (29.4%)	7 (6.4%)	16 (14.7%)	0 (0%)	2 (1.8%)	109
Total	6158 (82%)	4465 (59.5%)	2388 (31.8%)	3833 (51.1%)	634 (8.4%)	1150 (15.3%)	230 (3.1%)	1257 (16.7%)	7508

^1^ Total across all RV may exceed the number of samples submitted because a sample may be positive more than one category.

^2^ Midwest (Illinois, Indiana, Iowa, Kansas, Michigan, Minnesota, Missouri, Nebraska, Ohio, North Dakota, Wisconsin).

South-central (Oklahoma, Texas).

Southeast (North Carolina South Carolina).

Other-USA (South Dakota, Pennsylvania, Colorado, Arizona, Alabama, Arkansas, Florida, Kentucky, Tennessee, Utah, Virginia, Vermont, Wyoming).

Non-USA (Mexico and Canada).

**Fig 4:**
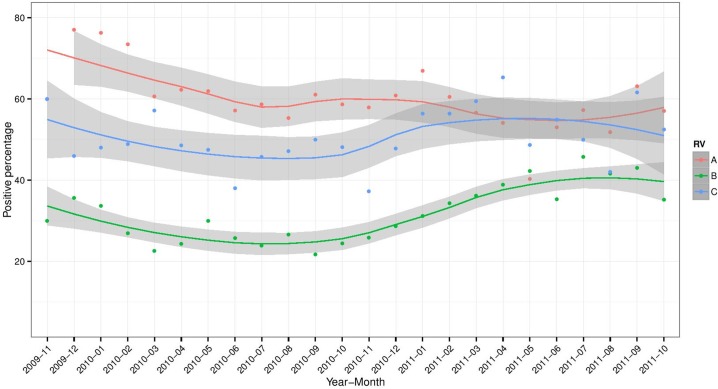
Percentages of positive RVA, RVB, and RVC samples by month. Red, green, and blue dots represent the percentage of positive RVA, RVB, and RVC samples, respectively. Time-series were smoothed by Locally Weighted Regression method, and the gray area represents 95% confidence interval for the fitted line.

To investigate the seasonality of RVA, RVB and RVC detection among swine diagnostic samples, the percentage of positive samples (observed and expected) were plotted over time (Fig 4). Overall, the expected percentage of positive samples was higher for RVA than for RVC and RVB. However, between April and July 2011, the expected percentage of RVA and RVC overlapped. In addition, the expected percentage of RVA detections decreased from 72% to 57% during the study period while the expected percentage of RVB positive samples increased from 33% to 40%. Nevertheless, the percentage of RVC detections remained relative stable (53% to 51%) throughout the study.

Since age as a continuous variable was not normally distributed (p-value <0.01), samples were categorized into five different age groups. Univariate logistics regression models were employed to determine if age and regions (Model 1 and 2, respectively) were risk factors for RV detection, and the RVA, RVB, RVC, RVAB, RVAC, and RVABC crude odds ratios (OR) were estimated (S2 Table). In Models 1 and 2, both age and regions were significant (p-value<0.001, except for RVAB in model 2, p-value = 0.042), indicating both variables should be in the same model. Thus, the 3L-MLMs (Models 3–5) were employed to partition and understand the variability of RV detection (Table 2). The 3L-MLMs contained a variation of model specifications, including fixed effects of region, random effects of age and regions, and the interaction between age and regions. Model 5, with age as fixed effect and random region and state effects as random effect, had the lowest Pseudo-AIC, indicating it was the preferred final model, and the COVTEST function verified the final model (p-value <0.001).

**Table 2:** Model selection for three-level mixed-effects logistic regression models.

Model	Model specification					AIC/Pseudo-AIC^1^						
	Fixed effect		Random effect									
	Age	Region	Interaction (Age:Region)	Region	States	RVA	RVB	RVC	RVAB	RVAC	RVBC	RVABC
1	✓					8916.7	8452.7	10162.0	4226.4	6294.5	1998.4	6041.7
2		✓				10051.9	9340.7	10353.7	4351.2	6417.6	2037.9	6732.7
3	✓	✓	✓	✓	✓	30891.0	31344.2	29334.9	37917.2	33945.2	NA	NA
4	✓	✓		✓	✓	30869.3	31291.9	29244.1	NA	33823.6	NA	35075.3
5^2^	✓			✓	✓	30846.2	31259	29224	37757.6	33775.5	44303.6	35011.2
COVTEST for Model 5						<0.001	<0.001	<0.001	<0.001	<0.001	<0.001	<0.001

^1^AIC for univariate models (Models 1 and 2) and Pseudo-AIC for three-level mixed-effects models (Models 3–5).

^2^Final model is indicated by lowest Pseudo-AIC.

NA means the models did not converge.

For the final model, the conditional odds ratios (cORs) of being positive to RV infection by age groups after adjusting for sample source variation were calculated (Table 3). The cORs for RVA and RVB were lower for pigs in the 1–3 days old compared to the other age groups. However, the odd of RVC detection in the 1–3 day age group was higher than in the 4–20 and the >55 day age groups (p<0.001). Furthermore, pigs in the 21–55 day age group had an increased odds for RV co-detections compared to pigs in the 1–3 day age group. The random effects for the region (γ^2^) and the states (τ^2^) were estimated from the assumptions of the final model to calculate the intra-class correlation coefficient of *ρ(region)* and *ρ(States*, *region)* to compare the variability within region and among states. The intra-class correlation coefficient of *ρ(States*, *region)* was greater than *ρ(region)* for RV detection, implying RV status was more similar within states than among states or within regions.

**Table 3:** The final model (age as fixed effect, with region and state as random effect) of three-level mixed-effects logistic regression.

Component	RVA	RVB	RVC	RVAB	RVAC	RVBC	RVABC
**Age Group (Fixed Effect)**	**Conditional odds ratio (95%CI)**						
1–3 days	-	-	-	-	-	-	-
4–20 days	1.96^c^ (1.66–2.316)	1.86^c^ (1.46–2.36)	0.53^c^ (0.46–0.63)	1.72^b^ (1.14–2.61)	1.26 (0.99–1.61)	0.91 (0.52–1.58)	3.80^c^ (2.40–6.04)
21–55 days	11.56^c^ (9.69–13.79)	8.06^c^ (6.43–10.09)	0.96 (0.82–1.12)	4.15^c^ (2.81–6.12)	2.33^c^ (1.86–2.93)	1.83^a^ (1.11–3.01)	19.40^c^ (12.46–30.20)
>55 days	3.65^c^ (3.04–4.38)	8.69^c^ (6.84–11.04)	0.55^c^ (0.47–0.66)	4.39^c^ (2.92–6.60)	0.9 (0.68–1.19)	3.64^c^ (2.20–6.01)	13.51^c^ (8.58–21.29)
**Random effects**	**Covariance estimates**						
Region, γ^2^ (SE)	0.032 (0.067)	0.06 (0.091)	-	-	0.021 (0.115)	0.044 (0.162)	0.074 (0.179)
States, τ^2^ (SE)	0.113 (0.063)	0.087 (0.065)	0.126 (0.051)	0.004 (0.010)	0.102 (0.075)	0.172 (0.122)	0.152 (0.103)
**Intra-class correlation**							
ρ(region)	0.009	0.017	-	-	0.006	0.013	0.021
ρ(States, region)	0.042	0.043	0.037	0.001	0.036	0.061	0.064
Pseudo-AIC	30846.23	31259.05	29224	37757.55	33775.54	44303.59	35011.2

^a^p-value < 0.05.

^b^p-value < 0.01.

^c^p-value < 0.001.

*ρ*(region) = γ^2^ / (γ^2^+τ^2^+π^2^/3), *ρ*(states, region) = (γ^2^ +τ^2^)/ (γ^2^+τ^2^+π^2^/3).

SE is standards of error of means.

## Discussion

To better understand the epidemiology of RV infection in pigs with enteric disease, 3L-MLMs were developed to estimate the association between RV detection by RRT-PCR and age in veterinary diagnostic samples. The effect of geographical location was incorporated to adjust the associations (conditional odds ratios) of RV detection among age groups. The detection of the different RV species was not evenly distributed within age groups or geographical regions. Understanding the distribution of RV infection among swine populations is important to develop better intervention practices to minimize the effect of RV infections on swine health.

The ecology of RV infections is complex, which has been demonstrated in different animal species [48]. Our results support those findings and indicate that the epidemiology of enteric diseases among swine populations is difficult due to the co-circulation of more than one RV species. While RV co-infections are common and complex, the ecology of RVA, RVB and RVC are different since they are not evenly distributed among pig age groups. In our study, older pigs (4–20, 21–55, and >55 days age groups) had higher cOR for RVA detection compared to piglets in the 1–3 day age group. Moreover, the cOR for RVA detection decreased in the > 55 day age group, which might be contributed to RV exposure and an increase of active immunity in the 21–55 day age group. Compared to RVA studies, RVC research is extremely limited. In our study, a higher proportion of RVC positive samples were present in 1–3 day old piglets, highlighting the significance of RVC on neonatal enteric disease. We hypothesize that the variability of RVC exposure in sows is correlated to the lack passive immunity protection, leaving the 1–3 day old piglets susceptible to a RVC infection. While sows are exposed to RVC via naturally planned exposure events (i.e. feeding RVC infected material to the sows) before farrowing, swine producers still report problems in preventing clinical disease associated with RVC infections in piglets. Hence, further studies are required to understand the development of maternal immunity to RVC, and its effect on preventing infection in piglets.

Multilevel mixed-effects logistic regression models are designed to handle hierarchical structure data sets with binary outcome for a dependent variable and independent variables Multilevel mixed-effects logistic regression models are very versatile and powerful, especially with large data set because inaccurate estimates may be generated if the hierarchical structure (multiple-demographic information) and source of variability is ignored [23]. Fixed-effect logistic regression models for states and regions increase the number of additional parameters, which is equal to the number of higher-level units minus 1 (j-1 for state levels and k-1 for region levels). If the number of parameters (states) is large, estimating the number of nuisance parameters is difficult, which may yield poor estimates [49]. In our data set, state (α_ij_ = α+ α_i_+W_ij_) and region (α_i_ = α+U_i_) effects were treated as random intercepts with specified probabilistic distribution, and the nuisance parameters were not estimated because the analysis provided conserve estimates for the state and region effects [49].

Unsurprisingly, our model indicated variability in RV detection among states. While RV detection was similar to within-regions but not similar among regions, different swine densities in North America may lead to less variability within each region. Furthermore, swine production systems could differ between regions, which may explain the differences among regions. In addition, wind, humidity and temperature vary by states and may affect RV infections in each swine regions, which were considered part of the regional level random effect. Under experimental settings, RV particles were aerosolized, which could be transported between farms by the wind [50]. In addition, transportation of pigs between states was also considered as a regional level random effect in our multilevel mixed-effect logistic regression models. Farm management and production systems (all-in all-out vs. continuous flow swine productions systems) can affect dynamics of viral transmission, infection, and evolution. Dewey and colleagues demonstrated farm management practices, including farm expansion, early weaning, and all-in all-out production affect the dynamics of RV infections [51]. Since the farm management information is lacking with sample submission, these factors were deemed as states level random effects (L2 random intercept) to encompass variations between farm management and production systems.

In summary, RV infections are a significant cause of diarrhea in swine. Determining the RV species associated with clinical disease and estimating the risk of RV infection over time will lead to better intervention tools to minimize the effect on swine health. Due to the large geography and different swine production regions within North America, 3L MLMs were used to adjust for variability in states and regions, and indicated RV status was more similar within states than between states or within region. Piglets in the 1–3 day old age group were less risky to RVA and RVB infection but more risky to RVC infection while associations in the older age group piglets were reversed. Our research indicates the swine RV epidemiology is complex in North America, but one thing is known, RV species are associated with different age groups and varied by regions.

## Supporting Information

S1 Table Descriptive statistics for number of positive samples to RVs by age group. (DOCX)

S2 Table Univariate logistic regression models for the risk factors of age and location. (DOCX)
