# Supplementary material for: Three-Level Mixed-Effects Logistic Regression Analysis Reveals Complex Epidemiology of Swine Rotaviruses in Diagnostic Samples from North America
Source: PLoS One. 2016 May 4;11(5):e0154734. doi: 10.1371/journal.pone.0154734 (PMC4856330; doi:10.1371/journal.pone.0154734)
Supplement: S1 Table — (DOCX) [file pone.0154734.s001.docx]

**S1 Table.** **Descriptive statistics for the number of positive samples to RVs by age group.**

| **Age group** | **Rotavirus Positive Samples, N (%)** | | | | | | | | **Total^1^** |
| --- | --- | --- | --- | --- | --- | --- | --- | --- | --- |
|  | **Any RV** | **A** | **B** | **C** | **AB** | **AC** | **BC** | **ABC** |  |
| 1-3 days | 779 (81.7%) | 288 (31.0%) | 98 (10.3%) | 589 (61.7%) | 29 (3.0%) | 105 (11.0%) | 20 (2.1%) | 21 (2.2%) | 954 |
| 4-20 days | 1580 (73.7%) | 980 (45.7%) | 363 (16.9%) | 996 (46.5%) | 110 (5.1%) | 284 (13.3%) | 37 (1.7%) | 164 (7.7%) | 2144 |
| 21-55 days | 2354 (92.8%) | 127 (83.8%) | 1190 (46.9%) | 1491 (58.8%) | 294(11.6%) | 563(22.2%) | 83(3.3%) | 757 (29.8%) | 2538 |
| >55 days | 989 (82.0%) | 739 (61.2%) | 599 (49.6%) | 556 (46.1%) | 147(12.2%) | 115(9.5%) | 81(6.7 %) | 281 (23.3%) | 1207 |
| Unknown | 456 (68.6%) | 331 (49.8%) | 138 (20.8%) | 201 (30.2%) | 54(8.1%) | 83(12.5%) | 9 (1.4%) | 34 (5.1%) | 665 |
| Total | 6158 (82%) | 4465 (59.8%) | 2388(31.8%) | 3833(51.1%) | 634 (8.4%) | 1150 (15.3%) | 230 (3.1%) | 1257 (16.7%) | 7508 |

^1^Total across all RV exceeds the number of samples submitted because a sample may be positive more than one category.
