# Supplementary material for: Three-Level Mixed-Effects Logistic Regression Analysis Reveals Complex Epidemiology of Swine Rotaviruses in Diagnostic Samples from North America
Source: PLoS One. 2016 May 4;11(5):e0154734. doi: 10.1371/journal.pone.0154734 (PMC4856330; doi:10.1371/journal.pone.0154734)
Supplement: S2 Table — (DOCX) [file pone.0154734.s002.docx]

**S2 Tabl**e. **Univariate logistic regression models for the risk factors of age and location.**

| **Age group (Model 1)** | **Crude odds ratio (95%CI)** | | | | | | |
| --- | --- | --- | --- | --- | --- | --- | --- |
|  | **RVA** | **RVB** | **RVC** | **RVAB** | **RVAC** | **RVBC** | **RVABC** |
| 1-3 days | - | - | - | - | - | - | - |
| 4-20 days | 1.95^c^ (1.66-2.29) | 1.78^c^ (1.40-2.26) | 0.54^c^ (0.46-0.63) | 1.73 (1.14-2.67) | 1.24 (0.97-1.57) | 0.82 (0.47-1.42) | 3.68^c^ (2.32-5.83) |
| 21-55 days | 11.97^c^ (10.06-14.24) | 7.71^c^ (6.17-9.64) | 0.88 (0.76-1.03) | 4.18^c^ (2.83-6.17) | 2.31^c^ (1.84-2.88) | 1.58^a^ (0.96-2.59) | 18.88^c^ (12.15-29.34) |
| >55 days | 3.65^c^ (3.05-4.37) | 8.61^c^ (6.79-10.91) | 0.53^c^ (0.45-0.63) | 4.42^c^ (2.94-6.65) | 0.85 (0.64-1.13) | 3.36^c^ (2.04-5.52) | 13.48^c^ (8.57-21.20) |
| Unknown | 2.29^c^ (1.87-2.81) | 2.29^c^ (1.73-3.03) | 0.27^c^ (0.22-0.33) | 2.82^c^ (1.78-4.48) | 1.15 (0.85-1.57) | 0.64 (0.29-1.42) | 2.39^c^ (1.38-4.16) |
| Overall p-value^1^ | <0.0001 | <0.0001 | <0.0001 | <0.0001 | <0.0001 | <0.0001 | <0.0001 |
| **Region (Model 2)** |  | | | | | | |
| Other-USA | - | - | - | - | - | - | - |
| Midwest | 1.30^c^ (1.12-1.52) | 1.67^c^ 1.40-2.00 | 0.99 (0.85-1.15) | 1.17 (0.88-1.55) | 0.97 (0.79-1.19) | 2.56 (1.22-5.38) | 1.88^c^ (1.48-2.39) |
| South-central | 0.66^c^ (0.54-0.81) | 1.76^c^ (1.40-2.20) | 1.62^c^ (1.32-1.98) | 0.91 (0.61-1.34) | 0.62^c^ (0.46-0.84) | 3.03^c^ (1.67-5.51) | 1.65^c^ (1.22-2.23) |
| Southeast | 1.92^c^ (1.44-2.57) | 1.56^c^ (1.16-2.10) | 0.98 (0.75-1.29) | 1.42 (0.89-2.26) | 1.41 (1.00-1.98) | 2.56^a^ (1.22- 5.38) | 1.25 (0.82-1.89) |
| Non-USA | 1.36 (0.90-2.05) | 0.45^a^ (0.25-0.84) | 0.41^c^ (0.26-0.63) | 0.84 (0.37-1.89) | 0.9 (0.51-1.58) | NA | 0.16^a^ (0.04-0.65) |
| Overall p-value | <.0001 | <.0001 | <.0001 | 0.0419 | <.0001 | <.0001 | <.0001 |

^a^p-value < 0.05; ^b^p-value < 0.01; ^c^p-value <0.001

Dash indicates reference group.

NA indicates co-detection of RVBC was not identified from the Non-USA region and then the models did not converge.

^1^LR χ2 test, testing global null hypothesis.
